# UGCG and the glycosphingolipid rheostat: a metabolic checkpoint governing immune activation and tumor immune evasion

**DOI:** 10.1038/s41392-025-02413-6

**Published:** 2025-09-03

**Authors:** Sumei Chen, Xiang Wang, Youssef Jounaidi

**Affiliations:** 1 Department of Radiation Oncology, Hangzhou Cancer Hospital, Hangzhou, Zhejiang China; 2 Zhejiang Key Laboratory of Zero Magnetic Medicine, affiliated Hangzhou First People’s Hospital, Westlake University School of Medicine, Hangzhou City, Zhejiang Province China; 3 Department of Anesthesia, Critical Care and Pain Medicine, Massachusetts General Hospital and Harvard Medical School, Boston, MA USA

**Keywords:** Translational immunology, Immunotherapy

In a recent study in *Cell*, Morrison et al.^1^ identified Ugcg and B4galt5 as super-enhancer–driven genes essential for glycosphingolipid synthesis in natural killer (NK) cells and cytotoxic T cells. Genetic deletion or pharmacologic inhibition of UGCG disrupted cytotoxic granules, induced apoptosis, and blocked NK and CD8⁺ T cell expansion during viral infection, underscoring the critical role of glycosphingolipid metabolism in lymphocyte identity and effector function.

The human genome contains over one million enhancers, but only a select few function as super-enhancers (SEs) that are large genomic regions densely occupied by transcription factors, chromatin remodelers, and coactivators such as Mediator driving high-level expression of genes critical for cell identity and function. Morrison et al.^1^ identified several key NK cell identity genes residing in SE loci, including *Ccl3*, *Ifnγ*, *Ifngr1*, *Runx1*, and *Prf1*. Unexpectedly, the top-ranked SE was found not in a canonical effector gene, but in *Ugcg*, the gene encoding UDP-glucose ceramide glucosyltransferase (UGCG), an enzyme catalyzing the first committed step in glycosphingolipid (GSL) synthesis by converting ceramide to glucosylceramide (GlcCer), which is then converted to lactosylceramide (LacCer) by B4GALT5, with B4GALT6 acting redundantly.

The authors demonstrated that GSL metabolism, specifically the simple GSL branch, is selectively essential for NK cell development and CD8⁺ T-cell cytotoxic function. Deletion of *Ugcg* early in hematopoiesis abolished NK cell generation without affecting other immune lineages. Pharmacologic inhibition of UGCG using ibiglustat resulted in NK cell apoptosis, loss of cytotoxic granules, impaired expansion during viral infection, and disrupted granule integrity. Notably, B4galt5, also residing in an NK-specific SE locus, was equally essential; its product, LacCer, rescued NK cell viability in the face of UGCG inhibition. In contrast, complex GSLs such as asialo-GM1 were dispensable for NK survival and function. In CD8⁺ T cells responding to viral infection, both *Ugcg* and *B4galt5* were upregulated in parallel with cytotoxic genes, and their loss prevented the expansion of antigen-specific T cells. Thus, this study highlights LacCer as the central, indispensable metabolite in the GSL pathway for cytotoxic lymphocyte viability and function. LacCer can be further converted into gangliosides like GM3 (via ST3GAL5) and onward to GD3 and GT3 (via sialyltransferases such as ST8SIA1 and ST8SIA3).

Morrison et al.^1^ found LacCer to localize to cytolytic granules and the plasma membrane, where it functions beyond structural support to enable immune execution. As a critical component of lipid rafts, it facilitates recruitment of scaffolding proteins, Src-family kinases, and actin regulators to the immunological synapse. It supports receptor clustering and cytoskeletal alignment essential for granule convergence, maturation, and polarized release. Morrison et al. showed that under UGCG inhibition, NK cells exhibited fewer and morphologically aberrant cytotoxic granules, impaired calcium flux, and failed to form stable synapses with target cells. Although surface expression of adhesion molecules like CD2 remained intact, their recruitment to the synapse was defective, halting both activating and inhibitory signaling. These findings emphasize that UGCG-driven LacCer production is necessary for immune synapse formation and granule release, positioning LacCer as a metabolic linchpin for NK cell cytotoxicity. Genetic dissection of the GSL pathway confirmed LacCer’s unique role: while NK cells persisted in mice lacking complex gangliosides (*B4galnt1*^−/−^), deletion of *B4galt5* and *B4galt6* mirrored the NK cell loss observed in *Ugcg* knockouts.

Intriguingly, deletion of *St3gal5*, the enzyme converting LacCer to GM3, did not impair NK cells but instead increased their frequency. Here, we hypothesize that deletion of *St3gal5* may offer a relief from downstream ganglioside-mediated immunosuppression, since tumor-associated gangliosides such as GM3, and GD3, are known to mask antigens,^2^ control the TCR repertoire,^3^ inhibit Fc receptor expression on monocytes and macrophages, leading to suppressed cytotoxicity, by promoting Treg development, and dampening dendritic cell activity. Thus, the loss or inhibition of *St3gal5* could preserve LacCer and enhance immune activation. Furthermore, we believe it is possible that the loss of ST8SIA1/3 may further block the generation of immunosuppressive gangliosides further enhancing NK cells. Thus, this study reveals a lipid rheostat governed by UGCG, where the balance between LacCer and its complex immunosuppressive derivatives modulates immune cell survival, homeostasis, and effector potential.

Tumor cells exploit UGCG and downstream GSL enzymes to produce immunosuppressive gangliosides like GM3 and GD3. This pathway supports tumor immune evasion by masking and blocking the interactions of membrane proteins.^2^ The therapeutic vulnerability of this axis was highlighted in another recent study^3^ where UGCG inhibition (via eliglustat) synergized with anti–PD-1 therapy in tumor-bearing mice. Consistent with this duality, eliglustat treatment in tumor-bearing mice led to (1) Reduced tumor ganglioside levels and increased exposure of the major histocompatibility complex and tumor antigen peptides (2) Enhanced CD8⁺ T cell infiltration and (3) Synergistic tumor control when combined with anti–PD-1. However, at higher doses, eliglustat impaired T cell function,^3^ mirroring the deleterious effects of UGCG knockout or high-dose ibiglustat as observed in Morrison’s study. These contrasting outcomes underscore a central principle: UGCG is a double-edged sword, required in immune cells function, yet hijacked by tumors for immune escape.

UGCG supports tumor survival through two distinct mechanisms. First, apoptosis regulation: by converting ceramide to GlcCer, UGCG prevents ceramide-induced ER stress and apoptosis.^4^ Second, multidrug resistance (MDR): GlcCer and downstream gangliosides modulate membrane fluidity and facilitate the localization of efflux transporters such as MDR1 in lipid rafts, thereby reducing chemotherapy efficacy. Across cancer models, UGCG overexpression correlates with poor chemotherapy response, and its inhibition can restore drug sensitivity.^5^ Thus, UGCG acts as both an immune checkpoint and a chemoresistance engine, making it a uniquely strategic metabolic target.

**Fig. 1:**
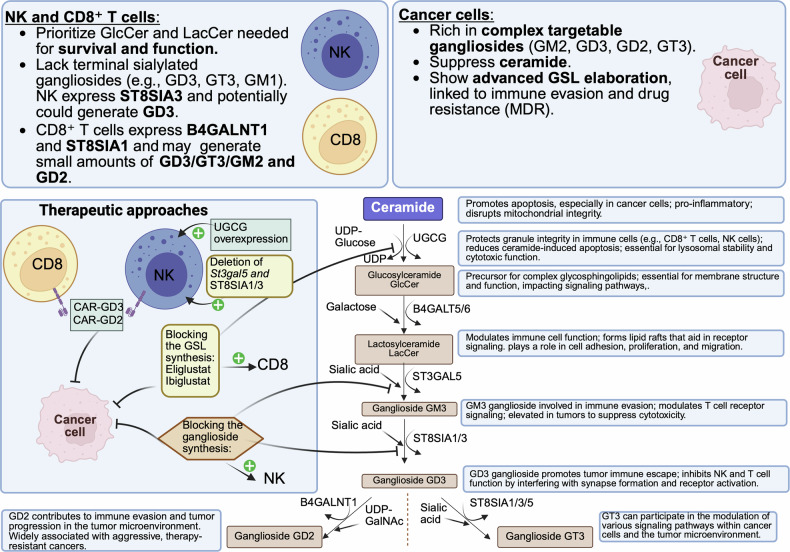
Divergent glycosphingolipid (GSL) metabolic profiles in immune and cancer cells highlight therapeutic targets. Cancer cells upregulate complex gangliosides (GM3, GD3, GD2, GT3) via ST3GAL5 and ST8SIA1/3, promoting immune evasion, drug resistance, and suppression of ceramide-induced apoptosis. In contrast, NK and CD8⁺ T cells depend on early GSLs like GlcCer and LacCer for granule integrity, lysosomal stability, and cytotoxic function. While NK cells naturally lack terminal gangliosides, making ganglioside inhibition (e.g., ST3GAL5/ST8SIA1/3 deletion) potentially enhancing to their function, CD8⁺ T cells may be enhanced by moderate GSL inhibition affecting mostly cancer cells or protected by UGCG overexpression. These differences suggest tailored approaches: targeting ganglioside synthesis to boost NK function and inhibit tumors, while protecting or engineering NK and CD8⁺ T-cells to resist GSL-targeting therapies. Therapeutic strategies include glycosyltransferase inhibition (eliglustat, ibiglustat), CAR-T cells against GD2/GD3, and genetic manipulation of UGCG or sialyltransferases. Illustration generated with BioRender.com

These findings suggest a new therapeutic opportunity: rather than globally suppressing UGCG, it may be more effective to strategically reprogram or selectively inhibit the glycosphingolipid pathway (Fig. 1), depending on cell context. Indeed, in addition to NK and CD8 T-cells, ILC1s also have high expression of *Ugcg* and *B4galt5*. Therefore, it is important to understand UGCG impact on ILC1 cytolytic activity. In tumors, intermediate-dose eliglustat or next-generation UGCG inhibitors could reduce immunosuppressive gangliosides (e.g., GM3, GD3) while preserving LacCer in immune cells. Blocking ST3GAL5 and probably ST8SIA1/3 could allow LacCer accumulation while preventing the formation of GM3 and GD3, thereby disarming tumor-driven immunosuppression without depriving immune cells of essential lipids. This requires precise dose titration and could be guided by metabolic biomarkers such as the LacCer:GM3/GD3 ratio or enhancer accessibility at the UGCG locus in different cell types. Meanwhile, in effector immune cells like NK and CD8⁺ T cells, enforced UGCG expression, via ex vivo engineering, CRISPRa activation, or targeted delivery of LacCer analogs, may help restore synapse integrity and cytotoxicity in immunosuppressive environments. Therefore, UGCG and its branches emerges as a tunable metabolic checkpoint governing immune activation and tumor immune evasion.
